# Catching up but still miles behind—a patient registry for otoferlin

**DOI:** 10.1038/s12276-024-01247-6

**Published:** 2024-06-03

**Authors:** Barbara Vona, Bernd Wollnik, Nicola Strenzke, Tobias Moser

**Affiliations:** 1 Institute of Human Genetics, University Medical Center Göttingen, Göttingen, Germany; 2 Institute for Auditory Neuroscience and InnerEarLab, University Medical Center Göttingen, Göttingen, Germany; 3 Cluster of Excellence “Multiscale Bioimaging: from Molecular Machines to Networks of Excitable Cells” (MBExC), University of Göttingen, Göttingen, Germany; 4 Department of Otolaryngology, University Medical Center Göttingen, Göttingen, Germany; 5 Auditory Neuroscience and Synaptic Nanophysiology Group, Max Planck Institute for Multidisciplinary Sciences, Göttingen, Germany

**Keywords:** Diseases, Genetics

The approval of investigational drugs for five adeno-associated virus-based gene replacement therapies marked a historic milestone in the journey to treat the first hereditary form of hearing impairment, auditory synaptopathy, caused by deleterious variants in the otoferlin gene (*OTOF*, DFNB9)^1^. Three of these approvals were granted by the U.S. Food and Drug Administration and European Medicines Agency in 2022/2023 and represented pivotal advancements, paving the way for clinical trials by one French-based and two American-based companies. In late 2023, the earliest results from the first-in-human otoferlin trial were presented at the European Society of Gene and Cell Therapy meeting in Brussels, Belgium (https://www.esgct.eu/) by Dr. Shu Yilai and colleagues from China, making headlines in the press (https://www.technologyreview.com/2023/11/01/1082740/china-gene-therapty-deafness-hearing/). This pioneering work, published in January 2024^2,3^, added substantial knowledge to the unfolding narrative, which also includes a recent press release from Akouos describing a successful one-month follow-up in a single treated patient (https://www.genengnews.com/topics/translational-medicine/gene-therapy-restores-hearing-in-11-year-old-after-just-one-month/). While early trial reports are cautiously optimistic, offering promising outcomes on safety and efficacy to date, open questions remain, including the durable safety and efficacy of these therapies in humans, as well as the implementation of harmonized and appropriate outcome measures. As we anticipate the development of efficient hearing-restoring *OTOF* gene therapies, we describe the inception of the first patient registry for patients with otoferlin-related auditory synaptopathy and the first gene-specific registry for an isolated form of hearing loss (NCT05946057), drawing inspiration from the existing registries for syndromic forms of hearing loss. This way, we mark yet another step forward in the evolving landscape of clinical trials on otoferlin-related auditory synaptopathy.

Hearing impairment is the most common sensory deficit in humans, affecting nearly 500 million individuals to a disabling extent^4^. Approximately 1–2 per 1000 newborns are diagnosed with hearing impairment, with a genetic cause in up to 80% of individuals^5,6^. Human genetics studies have identified several hundred genes associated with hearing impairment, and otoferlin ranks among the most common genetic causes^7^.

While hearing impairment has emerged as a collectively common yet heterogeneous disorder, the classification of hundreds of distinct genetic forms of hereditary hearing impairment as rare, individual diseases presents a unique challenge and contributes to an extraordinary burden on a few individuals dispersed across the world. The nature of rare diseases often subjects patients to a geographical lottery when trying to engage, for example, research participation or access to evolving treatment modalities that may one day include inner ear therapeutics^8^. Patient registries have emerged as a first step in understanding the disease, although they remain an underutilized resource to support research into pathogenesis, therapy, and longitudinal natural history studies, and can serve as a tool to recruit participants for clinical trials^9^.

An otoferlin patient registry linked to researchers who are passionate about otoferlin-related auditory synaptopathy should be viewed as a part of the infrastructure for inner ear therapeutics that is equally as important as development, diagnostics, regulatory approval pathways, distribution, marketing, and financing. Registries serve as a key part of the research process, which eventually relies on identifying sufficient patients for collaborative partnerships. The treatment of a single patient using gene therapies can cost hundreds of thousands or even millions of dollars. The approval of a drug by regulatory agencies such as the U.S. Food and Drug Administration does not mean that governments and insurance providers are likely to reimburse patients for that drug. Nevertheless, the real-time data amassed from registries offer a more detailed response to the initial inquiry faced by those seeking funding for preclinical research or determining the next target for investment: “What is the market?” Answering this question involves estimating the potential number of patients who could derive benefits from a particular treatment. Additionally, this data could influence reimbursement decisions, offering valuable insights that might be most effectively obtained through registry-based collection methods^10^.

We hope the registry will contribute to addressing more of the “known unknowns”, such as genotype–phenotype correlations and how hearing may change over time. Once therapies are available, this registry will be crucial in tracking data longitudinally in as many patients as possible for as long as possible. The total number of otoferlin patients is still a rough estimation limited to only a few countries. We also intend to use the registry as a bridge to invite exchanges with patient advocacy groups, which build on social media and other platforms.

It is surprising that a similar registry was not established earlier and that gene-specific registries for other inherited forms of isolated hearing loss are lacking. While we acknowledge the commendable efforts of general registries that consolidate all conceivable genetic data, there is an irreplaceable aspect to having a direct link with research groups interested in utilizing the data. Unless the goal is to inform national rare disease strategies, we anticipate more impactful outcomes by matching patients with enthusiastic teams interested in focused questions. We aspire to be part of the solution in the growing emergence of additional gene-specific registries to address crucial gaps, allowing for the exploration of natural history and establishing connections with patients. These infrastructures represent integral components in the complex equation needed to navigate the development and perfection of inner ear therapeutics.
